# Pro-angiogenic Role of Danqi Pill Through Activating Fatty Acids Oxidation Pathway Against Coronary Artery Disease

**DOI:** 10.3389/fphar.2018.01414

**Published:** 2018-12-04

**Authors:** Shihong Jiao, Binghua Tang, Yong Wang, Chun Li, Zifan Zeng, Lixia Cui, Xuefeng Zhang, Mingyan Shao, Dongqing Guo, Qiyan Wang

**Affiliations:** ^1^School of Life Sciences, Beijing University of Chinese Medicine, Beijing, China; ^2^Modern Research Center for Traditional Chinese Medicine, Beijing University of Chinese Medicine, Beijing, China; ^3^School of Traditional Chinese Medicine, Beijing University of Chinese Medicine, Beijing, China; ^4^State Key Laboratory of Bioactive Substances and Function of Natural Medicine, Institute of Materia Medica, Peking Union Medical College and Chinese Academy of Medical Sciences, Beijing, China

**Keywords:** DQP, angiogenesis, CAD, fatty acids oxidation, CPT1A, CD36

## Abstract

Coronary artery disease (CAD) is one of the leading causes of deaths worldwide. Energy metabolism disorders, including a reduction in fatty acids oxidation and upregulation of glycolysis pathway, are involved in the process of CAD. Therapeutic angiogenesis has become a promising treatment for CAD. Traditional Chinese medicines, such as Danqi Pill (DQP), have been proven to be effective in treating CAD in China for many years. However, the pro-angiogenic effects of DQP based on fatty acids oxidation are still unknown and the mechanism is worthy of investigation. In this study, left anterior descending (LAD) coronary artery was ligated to induce the CAD models *in vivo*, and cardiac functions were examined using echocardiography. Human umbilical vein endothelial cells (HUVEC) were subjected to H_2_O_2_-induced oxidative stress *in vitro*. The effects of DQP on CAD rat models and *in vitro* HUVEC were detected. Our results showed that DQP had cardio-protective effects in rat model. The intensity of capillaries in the marginal area of infarction of the rat heart was increased remarkably in DQP group, and the expression of PPARα and VEGF-2 were increased. The key enzymes involved in the transportation and intake of fatty acids, including CPT1A and CD36, both increased. In H_2_O_2_-induced endothelial cells injury models, DQP also showed protective roles and promoted capillary-like tube formation. DQP up-regulated key enzymes in fatty acids oxidation in H_2_O_2_-treated HUVEC. In addition, inhibition of CPT1A compromised the pro-angiogenic effects of DQP. In conclusion, fatty acids oxidation axis PPARα-CD36-CPT1A was involved in the pro-angiogenic roles of DQP against CAD. Cardiac CPT1A may serve as a target in therapeutic angiogenesis in clinics.

## Introduction

Coronary artery disease (CAD), also known as ischemic heart disease, is one of the leading causes of deaths worldwide. It caused 8.9 million deaths in 2015 and has become a large economic burden to the medical community (Boden et al., 2015). Stenosis or obstruction of the coronary arteries causes inadequate blood supply to the myocardium that further leads to myocardial ischemia, cellular necrosis, and heart failure. Conventional pharmaceutical treatments for CAD patients include nitroglycerin, statins, β-blockers, and calcium channel blockers (Conti, 2011; Bauters et al., 2014; Boden et al., 2015). Despite of the great efforts made in understanding and treating CAD, the therapeutic effects are still limited. Therefore, investigating novel methods for the treatment of CAD is an ongoing endeavor in the medical community.

Angiogenesis is the process of sprouting new blood vessels from pre-existing vasculature and is finely balanced and controlled. Therapeutic angiogenesis offers promise as a novel treatment for ischemic heart disease, especially for patients who could not tolerate current methods of revascularization. The goal is to relieve symptoms of ischemic heart disease and to protect heart functions by increasing blood supply to the ischemic region (Rosinberg et al., 2004). The main driver of angiogenesis is the arrangement of endothelial cells (EC) in tip and stalk cells. Vascular Endothelial Growth Factor (VEGF) and Notch signaling pathways are vital for tip cell differentiation (Adams and Alitalo, 2007). EC stay in quiescent state under normal conditions. The switch from a quiescent to a proliferative state is not only governed by genetic signaling cascades, but also accompanied with a “metabolic switch” in EC. It was recently found that metabolism was an essential regulator of angiogenesis (Pike et al., 2011; Debock et al., 2013; Schoors et al., 2014, 2015; Wong et al., 2016; Kim et al., 2017). Studies showed that angiogenic processes are dependent on the enhancement of metabolic pathways involving glucose, fatty acids, and glutamine. In particular, the role of fatty acid oxidation (FAO) in angiogenic process has been overlooked previously. Recent evidence showed that vessel sprout elongation relied on FAO which supplies the substrate for synthesis of deoxynucleotide triphosphates (dNTP) (Schoors et al., 2015). FAO involves multiple stages and the rate-limiting step is the import of fatty acids into mitochondria via CPT1A (Rosinberg et al., 2004). After being transported into mitochondria, fatty acids undergo β-oxidation, and produce acetyl-CoA, which further enters tricarboxylic acid (TCA) cycle. Entry of FA-derived acetyl-CoA sustained TCA cycle for the production of aspartate, which could be used for dNTP synthesis and essential for DNA replication in proliferating EC (Schoors et al., 2015). Studies showed that blockade of CPT1A leads to impaired vessel spouting (Pike et al., 2011). However, there are limited studies to investigate if activation of FAO by pharmaceutical methods could promote angiogenesis under ischemic conditions.

Controlling neovascularization is pivotal to treating CAD. Traditional Chinese medicines have been used to treat cardiovascular diseases for hundreds of years (Wong, 2014; Boden et al., 2015; Saleh et al., 2015; Al et al., 2016; Alali et al., 2016; Fardoun et al., 2017; Lu et al., 2018). Some of them have been shown to have angiogenic effects (Rosinberg et al., 2004). Danqi Pill (DQP), composed of Radix *Salvia miltiorrhiza* (Danshen) and Panax notoginseng (Sanqi), is among the most commonly prescribed TCM for heart diseases and has been shown to have cardio-protective effects (Liu et al., 2002). Salvianolic acids are the water extraction from Danshen and studies showed that Salvianolic acid A and B could increase blood vessel density in rats with myocardial infarction (Li et al., 2014; Yu et al., 2017). Our previous studies demonstrated that DQP could improve metabolism of heart under ischemic conditions. In particular, DQP could promote FAO by up-regulating transportation and uptake of fatty acids (Wang et al., 2015). The effects of DQP on angiogenesis are yet to be explored and whether DQP could promote angiogenesis through fatty acids oxidation pathway is worthy of investigation.

In this study, we will explore the effects of DQP on angiogenesis. The pharmacological mechanism of DQP on angiogenesis through fatty acids oxidation will be investigated by *in vivo* and *in vitro* studies. This study will provide new insights into the angiogenic mechanisms of TCM in treating ischemic diseases.

## Materials and Methods

### Materials

Danqi Pills (16120005) were purchased from TongrenTang (Beijing, China) and has a strict quality control by Pharmacopeia of the People’s Republic of China (Ministry of Health of the People’s Republic of China Pharmacopeia Committee, 2010) and definite clinical efficacy without known side effects. DQP is composed with the root of red-rooted salvia (*S. miltiorrhiza* Bge) and Panax notogenseng (Notoginseng Radix et Rhizoma). The fingerprint of DQP was analyzed by high-performance liquid chromatography (Supplementary Figure S1).

Medium 199 (M199, 10-060-CVR), matrigel (356231), and fetal bovine serum (FBS, 35-081-CV) were purchased from Corning (United States). Antibody against GAPDH (5174S) was purchased from Cell Signaling Technology (United States), antibodies against CD36 (ab64014), CPT1A (ab128568), VEGF-2 (ab10972), PPARα (ab3484) were obtained from Abcam (United States). CD31 (GB12063) was purchased from Servicebio (China). Secondary HRP-conjugated anti-mouse and anti-rabbit antibodies (Beijing TDY Biotech LTD, E009, and E011, respectively). Etomoxir (B1526025) was purchased from Aladdin (China). Trimetazidine (2010447) were purchased from Servier Pharmaceutical Company Limited (Tianjin, China). Calcinin (354216) was purchased from BD. Cell Counting KIT-8 was purchased from Japan (CCK-8, Dojindo Laboratories Inc., Kumamoto, Japan).

### Animals

The animal experiments were performed according to the Care and Use Guide of Laboratory Animals published by the National Institutes of Health (NIH Publications No. 85-23, revised 1996). All procedures involving animals were approved by the Animal Care Committee of Beijing University of Chinese Medicine. SD Rats (220 ± 10 g) used in the experiment were purchased from Sbef (Beijing) laboratory Animal Science and Technology Company (Beijing, China). Rats were housed in specific pathogen free (SPF) class animal house of Beijing University of Chinese Medicine with 12 days and night cycles in temperature 22 ± 2°C, humidity 50–60%.

### Ischemic Heart Model *in vivo*

Ischemic heart model was prepared as previously described (Wang et al., 2015). In brief, 1% pentobarbital (50 mg/kg) was injected into intraperitoneal for anesthesia. After tracheal intubation, thoracotomy was performed between the 4th and 5th intercostal of rats and left anterior descending (LAD) coronary artery was ligated. After closing the chest, 0.1–0.2 ml lidocaine and 0.1–0.2 ml furosemide were injected into the abdominutesal cavity. The sham rats underwent the same procedures except that the LAD was not ligated. After the rats resumed spontaneous breathing, the trachea was pulled out. According to a random number method, rats (that had undergone surgery and survived) were divided into a sham group, a model group, a DQP group, and a trimetazidine group on the second day, with ten rats in each group. Among them, rats in the DQP group received a daily oral gavage of DQP solution at dose of 1.5 mg/kg for 28 days. Rat in the positive control group was treated with a daily gastric trimetazidine solution at dose of 6.3 mg/kg for 28 days. Rats in the sham group and the model group had the same volume of saline. 28 days after surgery, 1% pentobarbital was injected into the abdominal cavity for anesthesia. The heart was harvested and frozen in liquid nitrogen.

### Echocardiographic Assessment of Cardiac Function

Echocardiography was used for measuring cardiac function. Parameters included ejection fraction (EF), left ventricular anterior wall; d (LVAW; d), left ventricular anterior wall; s (LVAW; s), Left ventricular end-diastolic diameter (LVED; d), left ventricular end-systolic diameter (LVED; s). The calculated Fractional shortening (FS%) is as follows: FS% = [(LVED; d-LVED; s)/LVED; d] × 100%.

### Western Blots

Proteins were extracted by Radio Immunoprecipitation Assay (RIPA) lysate (supplemented with a protein phosphatase inhibitor cocktail) (Beijing PuLilai Gene Technology Co., Ltd., Beijing, China, lot number: P-1260-1). The protein concentration was measured by using a protein quantification kit (Beijing PuLilai Gene Technology Co., Ltd., Beijing, China, lot number: P1511-1) and a Spectra Max i3x microplate reader (Molecular Devices, United States). Then the sample (50 μg, protein) was added to a 8% SDS-PAGE gel for electrophoresis, kept at 80 voltage for 30 min and then at 120 voltage for 75 min by using the PowerPac Universal Power Supply (United States Bio-Rad). The proteins on the gel were transferred to PVDF membrane (GE Healthcare, United States, 10600023) and electrophoresis was performed at 250 mA for 90 min. The membrane was incubated with primary and secondary antibodies and then treated with Amersham ECL primary immunoblot detection reagent (GE Healthcare, United States) for 1 min at room temperature. Membranes were exposed to a Molecular Imager ChemiDoc XRS+ system (Bio-Rad, United States) and the bands on the membranes were observed and analyzed by Image Lab software.

### Immunohistochemistry Analysis

Heart tissues were fixed in 4% paraformaldehyde for 72 h and then were embedded in paraffin and sectioned into 5 μm slices. Each group has four slides. The paraffin sections were subjected to haematoxylin-eosin (HE) and CD31 stainings. The comparison were made at similar location of the infarcted border heart tissues. The assessment was carried out by a researcher blinded to the interventions.

### EC Culture

Human umbilical vein endothelial cells (HUVEC) were purchased from Promocell (C12206, German) and cultured in the endothelial cells medium (C-22011, German) at 37°C and 5% CO_2_. EC were seeded 6000 cells/well in a 96-well plate. EC were stimulated with different concentration of DQP for 24 h and then treated with 50 μM H_2_O_2_ for 3 h to induce EC injury. 10 μl of CCK-8 was then added to each well. After 4 h, the optical density (OD) value of per well was determined with a microplate reader at 450 nm.

### *In vitro* Tube Formation Experiment

96 well plates were pre-coated with growth factor-reduced matrigel and the DQP-treated EC were seeded in well plates. After 2 h, EC were treated 50 μM H_2_O_2_ for 3 h. Then the diluted calcinin was added to the well for 10 min. The liquid was discarded. EC were washed twice with PBS and observed using an inverted fluorescence microscope. The results were analyzed by Image Pro-Plus software.

### Statistical Analysis

All data were presented as mean ± *SD*. Statistical analysis was performed with the SPSS program package (SPSS version 20.0) or GraphPad Prism 5. Statistical analysis was carried out using one way analysis of variance (ANOVA). The values of *P* < 0.05 were considered as statistically significant.

## Results

### DQP Showed Cardio-Protective Effects in Ischemic Heart Model

**FIGURE 1:**
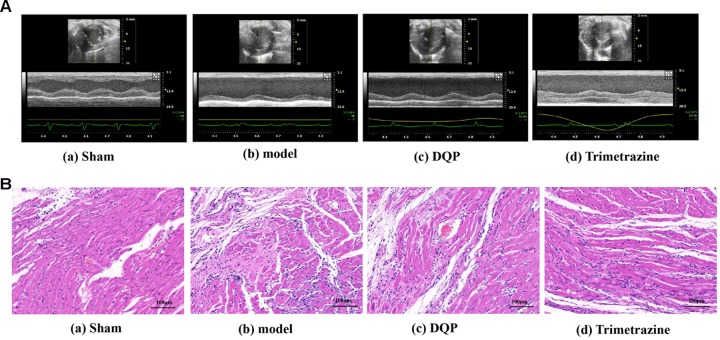
Danqi Pill (DQP) protected the cardiac function. **(A)** Representative images of echocardiography in sham, model, DQP, and Trimetazidine group. **(B)** Representative images of HE staining of the infarcted marginal area of the sham, model, DQP, and Trimetazidine group. Scale bar, 100 μM.

After ligation of LAD for 28 days, EF and FS were reduced by 60.5 and 71.8%, respectively, compared with sham group, indicating that ischemic model was successfully induced and cardiac functions were impaired in the model group. In DQP treatment group, EF was up-regulated by 44.0% and FS was up-regulated by 52.7% compared with model group. What’s more, LVID;s decreased in the DQP group (Figure 1A, Table 1). Positive control drug trimetazidine showed similar effects as DQP. In the sham group, the LV cardiac myocytes were arranged in an orderly pattern. In model group, myocardial cells in non-infarcted areas are hypertrophic, disordered, and infiltrated by inflammatory cells. DQP and Trimetazidine group significantly improved (Figure 1B). These results indicated that DQP had cardio-protective effects.

**Table 1:** Parameters of heart functions in four groups of rats.

Group	Sham	Model	DQP	Trimetazidine
EF	92.15 ± 3.248^∗∗^	36.39 ± 3.407^ 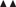 ^	52.38 ± 5.817^ 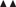 ^^∗∗^	55.82 ± 11.772^ 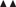 ^^∗∗^
FS	65.72 ± 5.109^∗∗^	18.53 ± 1.912^ 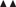 ^	28.30 ± 3.762^ 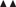 ^^∗∗^	30.85 ± 8.411^ 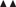 ^^∗∗^
LVAW;d	2.30 ± 0.438^∗∗^	0.81 ± 0.285^ 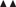 ^	1.31 ± 0.712^ 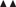 ^	1.95 ± 0.398^∗∗^
LVAW;s	3.94 ± 0.303^∗∗^	0.86 ± 0.325^ 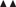 ^	1.76 ± 1.024^ 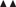 ^^∗^	2.13 ± 0.430^ 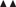 ^^∗∗^
LVID;d	5.89 ± 1.08^∗∗^	9.17 ± 2.461^ 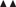 ^	9.18 ± 1.086^ 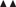 ^	8.45 ± 0.940^ 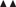 ^
LVID;s	2.07 ± 0.554^∗∗^	8.20 ± 0.822^ 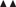 ^	6.55 ± 0.996^ 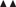 ^^∗∗^	5.76 ± 1.053^ 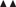 ^^∗∗^


^
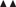
^*P* < 0.01, vs. sham group; ^∗^*p* < 0.05, ^∗∗^*p* < 0.01, vs. model group. EF, Ejection fraction; LVAW; d, left ventricular anterior wall; d; LVAW; s, left ventricular anterior wall; s; LVED; d, left ventricular end-diastolic diameter; d; LVED; s, left ventricular end-systolic diameter; s; FS, fractional shortening.

### DQP Promoted Angiogenesis in the Marginal Area of Infarction

**FIGURE 2:**
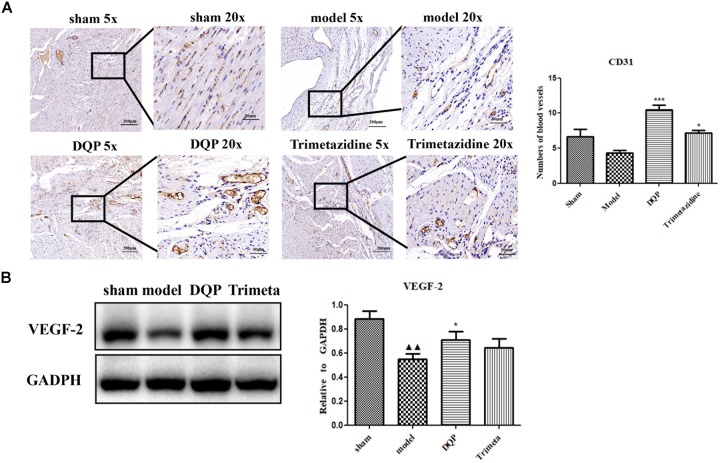
DQP could promote angiogenesis in the marginal area of infarcted heart. **(A)** Immunohistochemical staining of CD31. Representative microvascular micrographs were shown in the infarcted marginal area of the sham, model, DQP, and trimetazidine group (*n* = 4). DQP could increase the intensity of capillary vessels. **(B)** Western blot analysis of Vascular Endothelial Growth Factor (VEGF)-2 expression in the infarcted marginal heart tissues of the sham, model, DQP, and trimetazidine group. Quantitative analysis was presented in the graph (*n* = 4). Data were normalized to GADPH. ^
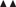
^*P* < 0.01 vs. sham group, ^∗^*P* < 0.05 vs. model group, ^∗∗∗^*P* < 0.001 vs. model group.

CD31 is a marker of angiogenesis and immunohistochemical staining showed that DQP could significantly increase the intensity of capillaries in the marginal area of infarction of the rat heart (Figure 2A). VEGF is the most potent and widely investigated proangiogenic growth factor. VEGF promotes angiogenesis by stimulating endothelial proliferation, migration, and capillary tube formation. The results showed that expressions of VEGF-2 were up-regulated by DQP treatment, indicating that DQP had angiogenic properties (Figure 2B). DQP also increased the expression of iNOS, decreased the expression of IL-1β and had no effect on LDAH (Supplementary Figures S3, S4).

### DQP Up-Regulated Fatty Acids Oxidation in the Ischemic Heart of Rats

**FIGURE 3:**
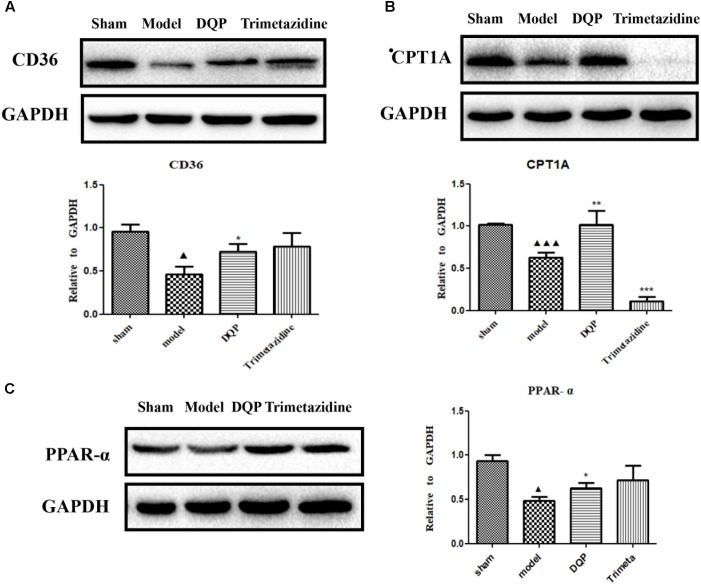
DQP reversed the lipid metabolism disorders in rat heart. **(A)** Western blot analysis of CPT1A expression in the infarcted marginal heart tissues in sham, model, DQP, and Trimetazidine group. Quantitative analysis was presented in the graph. Data were normalized to GADPH (*n* = 4). **(B)** Western blot analysis of CD36 expression in the infarcted marginal heart tissues in sham, model, DQP, and Trimetazidine group. Quantitative analysis was presented in the graph. Data were normalized to GADPH (*n* = 4). **(C)** Western blot analysis of PPAR-α expression in the infarcted marginal heart tissues in sham, model, DQP, and Trimetazidine group. Quantitative analysis was presented in the graph. Data were normalized to GADPH (*n* = 4). ^
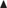
^*P* < 0.05 vs. sham group, ^
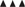
^*P* < 0.001 vs. sham group, ^∗^*P* < 0.05 vs. model group, ^∗∗^*P* < 0.01 vs. model group, ^∗∗∗^*P* < 0.001 vs. model group.

CD36, also known as fatty acid translocase, imports fatty acids into cells. CPT1A is the key rate-limiting enzyme during β-oxidation of fatty acids. It promotes the transportation of fatty acids from cytoplasm into mitochondria for oxidation. PPAR-α could promote the expression of CPT1A (Li et al., 2015). Expressions of these proteins were quantified by western blotting. DQP could significantly up-regulate expression of CPT1A in the marginal area of the infarcted heart (Figure 3A). Expression of CD36 increased in the DQP group, although the difference was not significant (Figure 3B). Expression of PPAR-α was also significantly up-regulated by DQP (Figure 3C). The data demonstrated that DQP could promote β-oxidation of fatty acids by regulating PPAR-a-CD36-CPT1A axis under ischemic conditions.

### DQP Protected EC Against H_2_O_2_-Induced Injury and Promoted Capillary-Like Tube Formation *in vitro*

**FIGURE 4:**
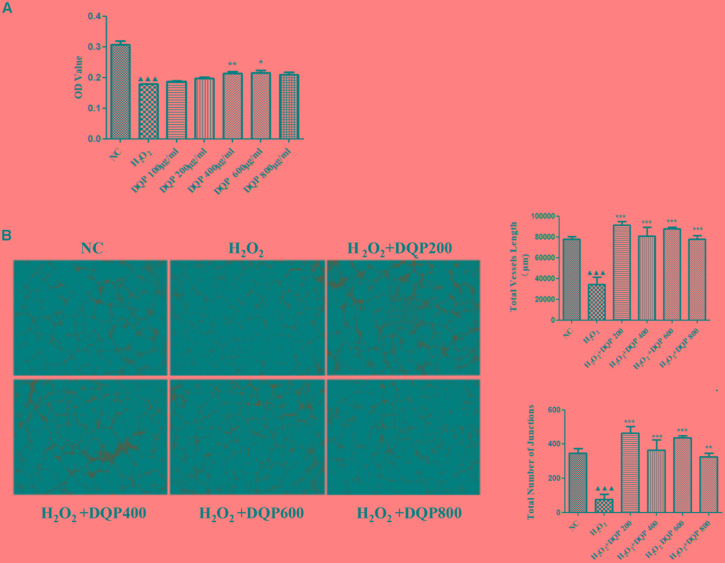
DQP protected EC from H_2_O_2_-induced injury *in vitro*. **(A)** The effects of different concentration of DQP on the endothelial cells (EC) proliferation induced by H_2_O_2_ (*n* = 8). Compared with cell injuried model group, treatment with 400 and 600 μg/ml DQP could up-regulate optical density (OD) by 19.0 and 20.1%, respectively. Data were presented as mean ± *SD*. **(B)** The effects of different concentration of DQP on the angiogenic ability of H_2_O_2_-treated Human umbilical vein endothelial cells (HUVEC) (*n* = 3). Treatment of HUVEC with DQP could significantly up-regulate vessel length and number of junctions formed by HUVEC.^
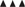
^*P* < 0.001 vs. NC group, ^∗^*P* < 0.05 vs. H_2_O_2_ group, ^∗∗^*P* < 0.01 vs. H_2_O_2_ group, ^∗∗∗^*P* < 0.001 vs. H_2_O_2_ group.

Endothelial cell injury model was induced by incubation with 50 μM H_2_O_2_. Different concentrations of DQP were applied to EC injury model. The effects of DQP on H_2_O_2_-induced cytotoxicity were detected by CCK8 assay and OD values were measured in different groups of cells. As shown in Figure 4A, DQP treatment (400 and 600 μg/ml) provided protective effects against H_2_O_2_-induced injury.

The angiogenic capability of HUVEC was assessed using capillary-like tube formation assay on growth factor-reduced matrigel. The formation of capillary-like tube network by EC was compromised by H_2_O_2_ treatment (Figure 4B). Capillary-like tubes with higher number of branching points and longer capillary tube lengths were observed in DQP treatment groups, suggesting that DQP possessed pro-angiogenic ability.

### DQP Up-Regulated Key Enzymes in Fatty Acids Oxidation in H_2_O_2_-Treated EC

**FIGURE 5:**
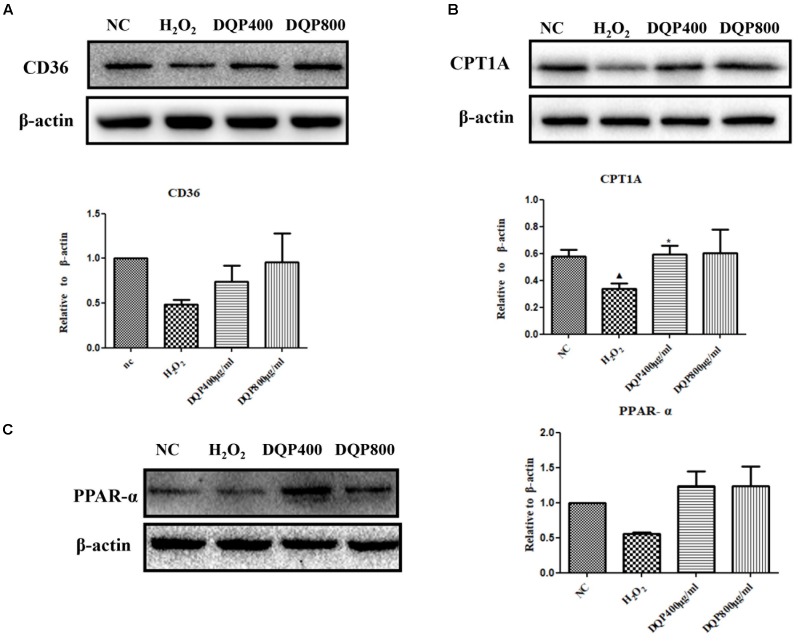
DQP regulated lipid metabolism disorders in H_2_O_2_-treated EC. **(B)** Western blot analysis of CPT1A expression in normal control (NC), H_2_O_2_, H_2_O_2_+DQP group (400 and 800 μg /ml). Quantitative analysis was presented in the graph. Data were normalized to ß-actin (*n* = 4). DQP could significantly up-regulated expression CPT1A by 40.5% compared with EC treated with H_2_O_2_. **(A)** Western blot analysis of CD36 expression in NC, H_2_O_2_, H_2_O_2_+DQP group (400 and 800 μg /ml). Quantitative analysis was presented in the graph. Data were normalized to ß-actin (*n* = 4). **(C)** Western blot analysis of PPAR-α expression in NC, H_2_O_2_, H_2_O_2_ +DQP group (400 and 800 μg/ml). Quantitative analysis was presented in the graph. Data were normalized to ß-actin (*n* = 4). ^
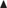
^*P* < 0.05 vs. NC group, ^∗^*P* < 0.05 vs. H_2_O_2_ group.

Effects of DQP on key enzymes in fatty acids oxidation pathways in H_2_O_2_-stimulated cells were examined. Treatment of EC with H_2_O_2_ reduced expressions of CPT1A, CD36, and PPAR-α. Incubation of EC with DQP (400 and 800 μg/ml) up-regulated expressions of CPT1A, CD36, and PPAR-α (Figure 5). The data suggested that DQP could promote fatty acids oxidation in endothelial cells by activating key enzymes in β-oxidation pathway.

### Inhibition of CPT1A Compromised the Pro-angiogenic Effects of DQP

**FIGURE 6:**
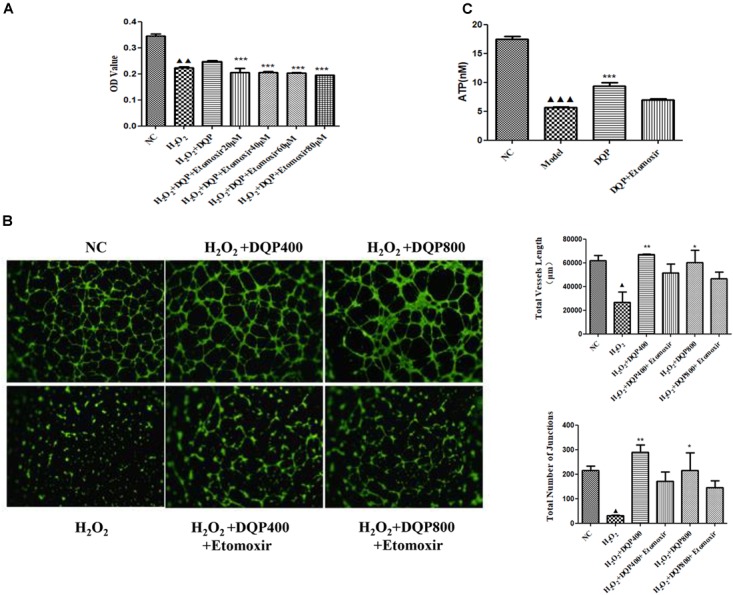
Inhibition of CPT1A impaired the protective and pro-angiogenic effects of DQP. **(A)**. HUVEC were stimulated with H_2_O_2_ (50 μM) and additionally co-treated with DQP (400 μg /ml). The proliferation of EC was detected by CCK8 assay. Different concentration of etomoxir, the inhibitor of CPT1A, could reverse EC proliferation induced by DQP (*n* = 8). **(B)** DQP promoted the angiogenesis of EC with the treatment of H_2_O_2_. Etomoxir could inhibit the pro-angiogenetic effects of DQP (*n* = 3). **(C)** DQP promoted the ATP production of EC with the treatment of H_2_O_2_. Etomoxir could inhibit the ATP production induced by DQP (*n* = 3). ^
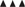
^*P* < 0.001 vs. NC group, ^
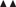
^*P* < 0.01 vs. NC group, ^
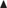
^*P* < 0.05 vs. NC group, ^∗^*P* < 0.05 vs. H_2_O_2_ group, ^∗∗^*P* < 0.01 vs. H_2_O_2_ group, ^∗∗∗^*P* < 0.001 vs. H_2_O_2_ group.

To investigate if DQP exerted angiogenic effects through fatty acids oxidation pathway, etomoxir, the specific inhibitor of CPT1A (Rupp et al., 2002), was used to treat H_2_O_2_-stimulated HUVEC with DQP. CCK8 assay showed that DQP protected EC against H_2_O_2_ induced cytotoxity. When endothelial cells were co-treated with etomoxir, proliferation of cells was reduced, as demonstrated by decreased OD value (Figure 6A). Furthermore, the pro-angiogenic ability of DQP was also compromised by blockade of CPT1A, as the capillary-like tubes formed by HUVEC were destructed by co-treatment with etomoxir (Figure 6B and Supplementary Figure S2). Meanwhile, the ATP production increased in response to DQP and inhibited by etomoxir (Figure 6C). These results suggested that DQP promoted tube formation ability of endothelial cells through fatty acids oxidation pathway.

## Discussion

In this study, we investigated the pro-angiogenic effects of traditional Chinese medicine DQP in the treatment of ischemic heart disease by *in vivo* and *in vitro* studies. The results demonstrated that DQP promoted angiogenesis through activating fatty acids oxidation pathway. Blockade of the rate-limiting enzyme CPT1A in FAO pathway compromised the angiogenic effects of DQP.

In patients with CAD, occlusion of coronary arteries often results in the development of collateral vessels which supply the ischemic tissue. However, this natural compensatory process of neovascularization is often not sufficient and revascularization procedures are often needed for patients with CAD (Khan et al., 2003). Therapeutic angiogenesis is a promising therapy for patients who are not amenable to current revascularization techniques. Gene therapy and cell-based therapy have been established a potential method to induce angiogenesis by preclinical studies and clinical trials (Bhang et al., 2014). There are, however, limitations of gene and cell therapies, such as low transmission efficiency, toxicity caused by viral vector, and inflammatory responses. Therefore, complementary treatment with pro-angiogenic drugs provides an alternative for therapeutic angiogenesis. A number of traditional Chinese medicine have been shown to have pro-angiogenic effects (Guo et al., 2018). The effects of DQP on angiogenesis haven’t been studied so far. We showed that DQP may exert cardio-protective effects through promoting revascularization under ischemic conditions. Ischemic heart model was induced by ligation of LAD in rats hearts as previously described (Wang et al., 2016). Consistent with previous reports, DQP showed cardio-protective effects as demonstrated by improved EF and FS. Trimetazidine is a clinically effective antianginal agents that regulates both fatty acid and glucose metabolisms and it was used as a positive control drug in this study (Kantor et al., 2000). Trimetazidine also had cardio-protective effects in ischemic heart model. CD31 is present on endothelial cells and is a marker of angiogenesis (Pusztaszeri et al., 2006). Immunohistochemical staining of CD31 showed that capillary densities in the marginal area of infarction were increased after treatment with DQP for 7 days, demonstrating the pro-angiogenic effects of DQP. DQP promoted expression of VEGF-2, which could regulate fatty acids uptake (Mehlem et al., 2016). *In vitro* studies also demonstrated that DQP could protect against H_2_O_2_-induced cellular injury and promote capillary-like tube formation by HUVEC. We further examined the pro-angiogenic mechanism of DQP and the role played by fatty acids oxidation during angiogenic process.

**FIGURE 7:**
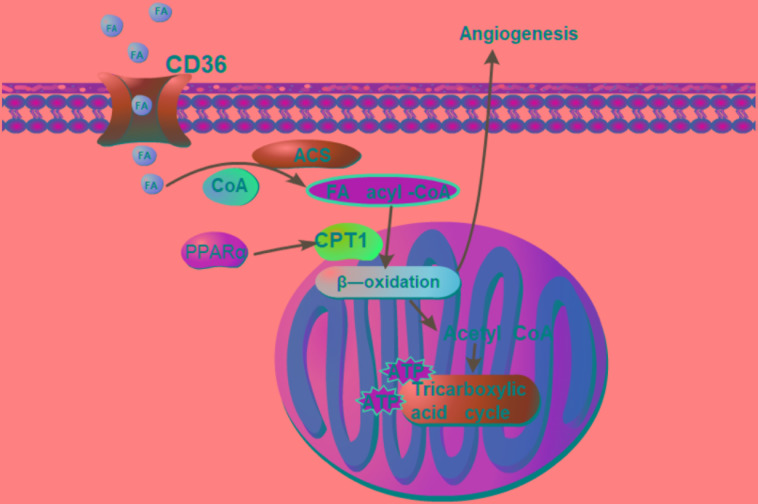
Summary of pro-angiogenic effects of DQP in CAD rats. Fatty acids oxidation axis PPARα-CD36-CPT1A is involved in the pro-angiogenic roles of DQP against CAD. The pro-angiogenic effects of DQP is probably mediated through up-regulating key enzymes in fatty acids oxidation pathway which is involved in proliferation of endothelial cells.

Fatty acids oxidation provides energy and substance for dNTP synthesis in endothelial cells. It involves several steps. Fatty acid transporter/CD36 transfers fatty acids into cytoplasm. CPT1A transfers the long chain fatty acids into mitochondria for β-oxidation and is the rate-limiting enzyme during FAO. Our results showed that DQP could up-regulate expressions of CPT1A and CD36 in the marginal area of infarction in the heart tissues and in H_2_O_2_-stimulated endothelial cells, suggesting that DQP could promote FAO by activating key enzymes under ischemic conditions. The importance of EC metabolism in regulating angiogenesis has only been unveiled by recent studies and EC metabolism is becoming increasingly recognized as a key determining mechanism of angiogenesis (Treps et al., 2016). EC mainly rely on glycolysis for the supply of energy. Even though fatty acids oxidation is able to generate more ATP per mole than glucose oxidation, FAO contributes minimal ATP production, but rather provides carbons for *de novo* nucleotide synthesis, thereby promoting proliferation of EC (Schoors et al., 2015). Studies showed that CPT1A deficiency reduces EC proliferation and *in vivo* angiogenic sprouting is impaired in mice lacking CPT1A in EC (Schoors et al., 2015). In our study, the results showed that pharmacological blockade of CPT1A with etomoxir impaired the pro-angiogenic effects of DQP, demonstrating the critical role played by FAO during angiogenesis. As DQP could up-regulate expression of CPT1A, we therefore hypothesize that DQP could promote angiogenesis through activating FAO pathway. Etomoxir, the inhibitor of CPT1A, is a promising drug to target pathological angiogenesis (Pike et al., 2011; Tyra et al., 2012). DQP, on the other hand, serves as an agonist of CPT1A and can be used to promote angiogenesis for ischemic diseases. As a limitation in the paper, haemodynamic measurements are necessary to evaluate the coronary flow in LV in the further.

Our study showed that DQP could promote angiogenesis in ischemic heart model. DQP could also promote the ability of HUVEC to form capillary-like tubes. The pro-angiogenic effects of DQP is probably mediated through up-regulating key enzymes in fatty acids oxidation pathway which is involved in proliferation of endothelial cells (Figure 7). Further studies should be performed to investigate the effective components of DQP that can act on FAO. Our study suggested that pharmacological activation of CPT1A might provide an alternative for therapeutic angiogenesis in the treatment of ischemic diseases.

## Author Contributions

SJ, BT, and YW contributed equally to this paper. DG and QW designed the experiments, wrote and modified the manuscript. SJ performed most of the experiments. BT and YW modified the manuscript. ZZ and CL helped the animal experiments. XZ and MS helped the cell experiments. All authors reviewed the manuscript.

## Conflict of Interest Statement

The authors declare that the research was conducted in the absence of any commercial or financial relationships that could be construed as a potential conflict of interest.

## Supplementary Material

FIGURE S1 Parameters of heart functions in four groups of rats. ^^*P* < 0.01 vs. sham group; ^∗^*P* < 0.05, ^∗∗^*P* < 0.01 vs. model group.

FIGURE S2 Etomoxir alone showed no effects on the tube formation. 20 μM Etomoxir alone showed no effects on the angiogenesis of endothelial cells (EC) (*n* = 3).

FIGURE S3 DQP regulated the LDHA expression in rat heart. Western blot analysis of LDHA expression in the infarcted marginal heart tissues in sham, model, DQP, and Trimetazidine group. Data were normalized to GADPH.

FIGURE S4 DQP regulated the iNOS and IL-1β expression in rat heart. **(A)** Western blot analysis of iNOS expression in the infarcted marginal heart tissues in sham, model, DQP, and Trimetazidine group. Data were normalized to GADPH (*n* = 4). **(B)** Western blot analysis of IL-1β expression in the infarcted marginal heart tissues in sham, model, DQP, and Trimetazidine group. Data were normalized to GADPH (*n* = 4). ^^*P* < 0.05 vs. sham group, ^∗^*P* < 0.05 vs. model group.
