# Supplementary material for: Pro-angiogenic Role of Danqi Pill Through Activating Fatty Acids Oxidation Pathway Against Coronary Artery Disease
Source: Front Pharmacol. 2018 Dec 4;9:1414. doi: 10.3389/fphar.2018.01414 (PMC6289089; doi:10.3389/fphar.2018.01414)
Supplement: FIGURE S1 — Parameters of heart functions in four groups of rats. P < 0.01 vs. sham group; ∗P < 0.05, ∗∗P < 0.01 vs. model group. [file Image_1.pdf]

Supplement 1

The chemical analysis of DQP by HPLC.

Grinding powder of DQP was weighed accurately (0.2 g) and placed into a 15 ml centrifuge tube containing 10 ml 50% aqueous methanol for 30 minutes in a ultrasonic cleaning (YH-200DH, Shanghai) at 40kHz. Following filtration by microporous filters (0.22μm) after cooling, the HPLC analysis was carried out on a Shimadzu HPLC (two LC-20AD solvent delivery units, a SIL-20A auto-sampler, a CTO-20A column oven, a SPD-M20A PDA detector, a DGU-20A degasser, and a CBM-20A controller). The standard ginsenoside Rb1, ginsenoside Rg1 and notoginseng R1 were purchased from Sigma Chemical Co. (St. Louis, USA) for the quality control according to Pharmacopoeia of the People's Republic of China(Ministry of Health of the People's Republic of China Pharmacopoeia Committee, 2010). The chromatographic separation was performed on an Agilent Zorbax SB C18 column (250 × 4.6 mm, 5 μm) without controlling of column temperature. Acetonitrile-water solution was used as the mobile phase for analysis. The flow rate was set at 1 ml/min and the wavelength was set at 203 nm. The elution condition was applied with a gradient program as follows: 0–15min, 3–15% acetonitrile solution; 15–30 min, 15–30% acetonitrile solution; 30–50 min, 30–40% acetonitrile solution; 50–55 min, 40% acetonitrile solution. 10 μl sample were injected into HPLC system for analysis. The HPLC results of DQP were presented in Supplementary Figure 1.

Supplementary Figure 1: Qualitative analysis on grinding powder of DQP.

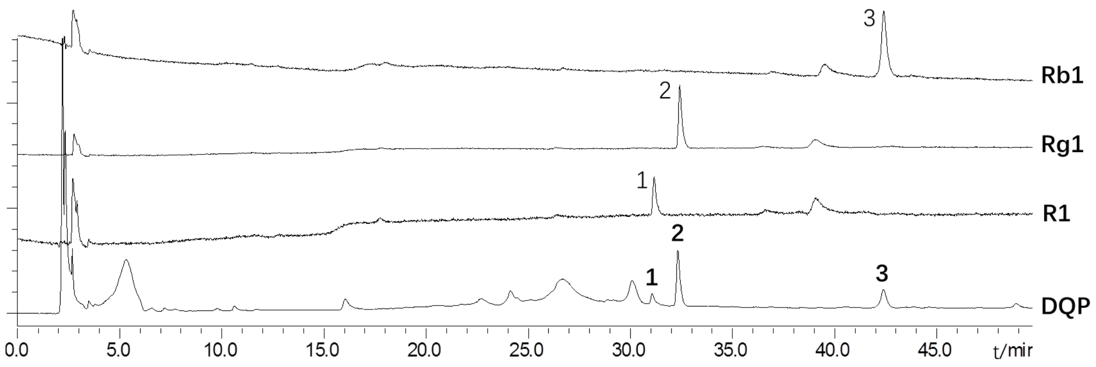

HPLC-PDA chromatograms numbered from 1 to 3 represented notoginseng R1, ginsenoside Rg1 and ginsenoside Rb1respetively which are applied as the standard of quality control according to China Pharmacopoeia (Ministry of Health of the People's Republic of China Pharmacopoeia Committee, 2010).
